# Health Care Professional Willingness to Treat Opioid Use Disorder vs Type 2 Diabetes in Primary Care

**DOI:** 10.1001/jamanetworkopen.2025.34680

**Published:** 2025-09-30

**Authors:** Lindsay Y. Dhanani, Daniel Brook, O. Trent Hall, Ji Eun Chang, Berkeley Franz

**Affiliations:** 1 Rutgers University School of Management and Labor Relations, Piscataway, New Jersey; 2 Department of Psychiatry, University of Colorado School of Medicine, Aurora; 3 Department of Psychiatry and Behavioral Health, The Ohio State University Wexner Medical Center, Columbus; 4 Department of Public Health Policy and Management, New York University, New York; 5 Institute to Advance Health Equity, Ohio University Heritage College of Osteopathic Medicine, Athens

## Abstract

**Importance:**

A substantial implementation gap remains for medications for opioid use disorder (MOUDs), which prevent overdose and associated morbidities. Primary care is an important setting for expanding access opioid use disorder (OUD) treatment.

**Objective:**

To compare primary care professional (PCP) willingness to treat OUD vs type 2 diabetes in primary care and to assess whether patient race is associated with shaping decisions to treat either condition.

**Design, Setting, and Participants:**

This cross-sectional study used a 2 (chief concern of OUD compared with type 2 diabetes) × 2 (Black compared with White race) experimental design. Physicians, nurse practitioners, and physician assistants practicing in primary care or a related discipline in Ohio were randomly assigned to each condition and completed assessments between March 1 and June 6, 2024.

**Exposure:**

Participants received fictitious patient profiles that were identical except that they either described the chief concern as OUD or type 2 diabetes and specified the patient’s race as Black or White.

**Main Outcomes and Measures:**

Clinical and attitudinal outcomes, including willingness to treat the patient in primary care, the amount of time the participant would spend with the patient, and perceptions of the likelihood of disease stabilization, were assessed using 5-point Likert scales (1 = extremely unlikely or strongly disagree, 5 = extremely unlikely or strongly agree). For OUD only, willingness to provide MOUD treatment and harm reduction were assessed.

**Results:**

A total of 375 participants provided complete data (mean [SD] age, 46.8 [13.6] years; 235 female [62.7%]), of whom 191 (50.9%) were physicians (MD or DO), 104 (27.7%) were nurse practitioners, and 80 (21.3%) were physician assistants. Participants viewed type 2 diabetes as more complicated to treat, viewed patients with type 2 diabetes as more responsible for their diagnosis, and had more empathy toward patients with OUD. Participants were significantly more willing to treat type 2 diabetes in primary care (mean [SD], 4.4 [0.1] vs 3.6 [0.1] in the OUD condition) and more likely to refer patients with OUD to a specialist (mean [SD], 4.4 [0.1] vs 3.3 [0.1] in the type 2 diabetes condition). No significant treatment differences were found by patient race. Participants were moderately likely to prescribe an MOUD to the hypothetical patient, with slightly higher willingness to prescribe naltrexone (mean [SD], 3.7 [1.2]) than buprenorphine (mean [SD], 3.4 [1.3]). The most strongly endorsed treatment was referral to an abstinence-based residential treatment organization (mean [SD], 3.9 [1.2]).

**Conclusions and Relevance:**

These findings suggest that PCPs have positive attitudes toward treating OUD yet more willingness to treat type 2 diabetes in primary care. Future implementation studies are needed to support MOUD prescribing in primary care.

## Key Points

**Question:**

Are primary care professionals (PCPs) more willing to treat opioid use disorder (OUD) or type 2 diabetes in the primary care setting?

**Findings:**

This cross-sectional study of 375 PCPs in Ohio found that participants viewed type 2 diabetes as more complicated to treat, viewed patients with type 2 diabetes as more responsible for their diagnosis, and had more empathy toward patients with OUD. However, participants were more willing to treat type 2 diabetes in primary care and more likely to refer patients with OUD to a specialist.

**Meaning:**

These findings suggest that future implementation work is needed to determine whether addressing the implementation climate, especially perceptions that primary care is compatible with OUD treatment, could improve treatment access and OUD outcomes.

## Teaser

This cross-sectional study compares primary care professional willingness to treat opioid use disorder (OUD) vs type 2 diabetes in primary care and assesses whether patient race is associated with decisions to treat either condition.

## Introduction

Highly effective medications for opioid use disorder (MOUDs) strongly reduce overdose risk, and because they also reduce opioid use, MOUDs are associated with less frequent infectious disease transmission. Despite its efficacy, MOUD access remains extremely limited.^1^ Leading medical societies have advocated for improving access to MOUDs in primary care because the primary care setting is less stigmatizing than formal addiction treatment programs and allows for other medical needs to be addressed.^2^ Recent policy changes have also emphasized expanding access within primary care, especially removing the decades-long X-waiver requirement, which mandated special training for buprenorphine prescribers. Unfortunately, deregulation has not meaningfully improved prescribing.^3,4^ A critical challenge to expanding access to MOUDs remains the willingness of primary care professionals (PCPs) to screen for and deliver evidence-based treatment for addiction, including MOUDs.

Barriers to providing opioid use disorder (OUD) treatment in the primary care setting have been extensively described in the literature. Limited time to manage complex conditions such as OUD and to prescribe an MOUD^5^; low confidence and training among PCPs^6^; and negative attitudes toward OUD treatment, including buprenorphine,^7,8^ persist. For example, studies have shown an association between negative attitudes, including perceived controllability of OUD, low empathy, and lack of previous positive experiences with patients with OUD, and willingness to treat this patient population in primary care.^9,10,11^

It is also not well understood whether PCPs view prescribing MOUDs as compatible with primary care. Perceived compatibility is part of the overall implementation climate, which shapes whether professionals feel supported to deliver an evidence-based intervention,^12^ such as an MOUD. One way to explore perceived compatibility is to compare willingness to treat OUD with willingness to treat other chronic diseases, such as type 2 diabetes, that are commonly managed in the primary care setting^13^ and have similar complexities.^14^ It also is not clear what determinants might shape perceived compatibility,^15^ including the degree to which the condition is perceived as controllable,^16,17^ the perceived complexity of treatment,^5^ and confidence managing each condition.^6^ Understanding these determinants is critical to providing implementation support.

Access to treatment for OUD and other chronic diseases in primary care may also be associated with patient-level social factors, including race. Individuals from minoritized racial groups receive lower-quality care, including longer wait times,^18,19,20^ less time with health care professionals,^21^ and less access to evidence-based treatments, including MOUDs.^22,23^

The primary aim of this study was to assess differences in PCP treatment willingness for patients with OUD vs type 2 diabetes. A secondary goal was to assess whether a patient’s race is associated with decisions to treat either OUD or type 2 diabetes in the primary care setting.

## Methods

### Study Population

This cross-sectional study recruited physicians and advanced practice professionals licensed to practice in the state of Ohio. The study was approved by the Ohio University Institutional Review Board, and all PCPs provided electronic informed consent before participation. This study followed the Strengthening the Reporting of Observational Studies in Epidemiology (STROBE) reporting guideline for cross-sectional studies.

Participants were eligible for inclusion if they were a physician, nurse practitioner, or physician assistant in a primary care–aligned field. Participants met inclusion criteria if they practiced in primary care or a related specialty that frequently cares for patients with OUD, including family medicine, internal medicine, addiction medicine, obstetrics and gynecology, infectious disease, emergency medicine, and psychiatry. For recruitment, we emailed all eligible PCPs in the included disciplines using the State Board of Medical Licensing roster. Because of the size of the database, we did not check whether individual email addresses were up to date. The database is maintained annually and indicates that PCPs were practicing in Ohio during the previous year. However, email addresses are given at the time of licensure and are not regularly updated; thus, calculating a valid response rate was not feasible.

We emailed participants in batches and sent 2 reminder emails approximately 3 and 7 days after the original email invitation. The email invitation sought perspectives on managing chronic disease in the context of primary care. Data were collected between March 1 and June 6, 2024. We set an a priori goal of recruiting 400 participants and closed the survey when we reached that goal. Participants were eligible for a raffle to win a $100 online shopping gift card. To maintain anonymity, participants were rerouted to a new survey to provide identifying contact information for the raffle.

### Procedures

We used a 2 (chief concern of OUD compared with type 2 diabetes) × 2 (Black compared with White race) experimental design in which participants were randomly assigned to read a patient profile that described the chief concern for the health care visit. The patient profiles were created by the study team and were identical except that they described the chief concern as either OUD or type 2 diabetes and identified the patient’s race as either Black or White. We included only Black and White as race options in the fictitious profiles because our study was only powered for a 2 × 2 design. Given the well-documented disparities in treatment received between Black and White individuals in the US, we opted to test differences between these 2 racial groups.^24^ Participants were asked to read the provided profile and evaluate their willingness to treat the patient in primary care.

### Key Variables

#### Clinical Outcome Intentions

Clinical outcomes included willingness to treat the patient in primary care, the amount of time the participant would spend with the patient, and perceptions of the likelihood of disease stabilization. Willingness to treat the patient was captured using 2 items that asked, “How likely are you to agree to treat this patient,” and, “How likely are you to refer this patient to a specialist?” Responses were coded on a 5-point Likert scale ranging from 1 (extremely unlikely) to 5 (extremely likely). The estimated time spent with the patient was provided in increments of 10 minutes. Finally, we measured the perceived likelihood of disease stabilization using a 5-point Likert scale, with responses ranging from 1 (extremely unlikely) to 5 (extremely likely).

For OUD only, we measured 6 additional treatment and harm reduction outcomes, including the likelihood of prescribing buprenorphine and naltrexone and the likelihood of referring the patient to an abstinence-only residential treatment facility. Harm reduction outcomes included the likelihood of checking with a patient about the need for naloxone, prescribing preexposure prophylaxis for HIV prevention, and referring the patient to a syringe service program. Responses for these treatment and harm reduction outcomes were coded on a 5-point Likert scale ranging from 1 (extremely unlikely) to 5 (extremely likely).

#### Attitudinal Outcomes

We measured controllability using a 4-item scale that assessed beliefs about whether patients are in control of or are responsible for their addiction. The scale was originally used to measure beliefs about people who inject drugs.^25^ A previous study adapted it to focus on patients with OUD and found evidence for reliability and validity with a different sample of PCPs.^11^ In this study, we presented participants who were assigned to the OUD condition with items assessing the perceived controllability of OUD, as well as adapted the same items for the type 2 diabetes condition. Responses were provided on a 5-point Likert scale ranging from 1 (strongly disagree) to 5 (strongly agree). Higher scores indicated higher perceived controllability.

We measured empathy using a 6-item scale developed by Batson et al.^26^ This scale was previously adapted in another study that found evidence of reliability and validity for measuring empathy toward people with OUD among a separate sample of PCPs.^9^ This scale contains a series of feelings that represent empathy (eg, sympathetic, compassionate, warm) and asked participants to indicate the extent to which they experience each of these feelings toward either people with OUD or type 2 diabetes. Responses were provided on a scale from 1 (not at all) to 7 (extremely).

#### Covariates

Positive contact, or the extent to which PCPs had contact with patients with OUD or type 2 diabetes that they appraised positively, was measured using 3 items developed by Plant and Devine.^27^ These items were previously adapted in another study that found evidence for reliability and validity for measuring how pleasant, rewarding, and positive respondents’ past experiences of working with people with OUD had been.^9^ We further adapted these items for this study to reflect contact with patients with type 2 diabetes. Responses were given on a 5-point Likert scale from 1 (strongly disagree) to 5 (strongly agree), with higher scores indicating more positive contact.

We also accounted for participant demographics associated with our study variables. Specifically, we measured sex (coded as male = 1 and female = 2), self-reported race (coded as White = 1 and American Indian or Alaska Native, Asian, Black, Native Hawaiian or Pacific Islander, biracial or multiracial, or prefer not to respond or to self-describe = 2), PCP type (physician, nurse practitioner, or physician assistant), and whether the participant worked in a Federally Qualified Health Center (yes or no).

### Statistical Analysis

We computed descriptive statistics to describe participant demographics and OUD treatment willingness among participants in the OUD condition. To analyze data across conditions, we conducted three 2 × 2 multivariate analyses of covariance (MANCOVAs) in which the factors were chief concern (OUD or type 2 diabetes) and patient race (Black or White) and the covariates were positive contact, participant sex, participant race, PCP type, and whether the participant worked in a Federally Qualified Health Center. We additionally assessed for any potential interactions between the 2 experimental conditions to examine whether there were any joint associations of chief concern and race. The first model included clinical outcomes as the dependent variables, the second model included perceived controllability and empathy as the dependent variables, and the third model included the 6 attributions for the chief concern as the dependent variables. We first examined whether the overall *F* test was significant, and if so, we then examined the *F* tests for each individual dependent variable and inspected the pairwise comparisons to determine where the significant differences (*P* < .05) arose. Herein, we present the results for the overall MANCOVA tests; the individual analysis of covariance results for each dependent variable included in the analyses; and the means, SEs, and mean differences between conditions for any significant findings. All analyses were conducted using SPSS, version 29 (IBM Corp).

## Results

### Participant Characteristics

A total of 409 participants responded to the survey, and 375 provided complete data and were included in the analytic sample (mean [SD] age, 46.8 [13.6] years; 235 female [62.7%] and 140 male [37.3%]; 2 self-identified as American Indian or Alaska Native [0.5%], 42 as Asian [11.2%], 10 as Black [2.7%], 1 as Native Hawaiian or Pacific Islander [0.3%], and 304 as White [81.1%] race; 7 self-identified as biracial or multiracial [1.9%] and 9 as preferring not to respond or preferring to self-describe their race [2.4%]). A total of 191 participants (50.9%) were physicians (MD or DO), 104 (27.7%) were nurse practitioners, and 80 (21.3%) were physician assistants. Participant board certifications were 206 (54.2%) in family medicine, 123 (32.4%) in internal medicine, 8 (2.1%) in obstetrics and gynecology, 41 (10.8%) in emergency medicine, 9 (2.4%) in psychiatry, 11 (2.9%) in addiction medicine, 7 (1.8%) in infectious diseases, and 10 (2.6%) in pain management. A total of 83 participants (22.1%) reported working in a Federally Qualified Health Center. Participants worked a mean (SD) of 39.6 (14.8) hours per week. There were no significant differences in PCP demographics between intervention conditions at baseline.

### Descriptive Results for OUD Treatment Willingness

Participants in the OUD condition were moderately likely to prescribe MOUDs, with a slightly higher willingness to prescribe naltrexone (mean [SD], 3.7 [1.2]) than buprenorphine (mean [SD], 3.4 [1.3]). The most strongly endorsed treatment was referral to an abstinence-based residential treatment organization (mean [SD], 3.9 [1.2]). In terms of harm reduction, participants were most likely to check with the patient about the need for naloxone (mean [SD], 4.5 [0.8]). Participants had a much lower willingness to refer patients to syringe exchange programs (mean [SD], 3.4 [1.3]) or prescribe preexposure prophylaxis (mean [SD], 3.1 [1.2]). None of the treatment decisions varied significantly based on the patient’s race (Table 1).

**Table 1.** Opioid Use Disorder Response Scores by Patient Race

Item	Response score, mean (SD)^a^		
	Overall	Patient race	
		White	Black
Willingness to prescribe buprenorphine	3.4 (1.3)	3.5 (1.3)	3.3 (1.3)
Willingness to prescribe naltrexone	3.7 (1.2)	3.8 (1.2)	3.6 (1.3)
Recommend abstinence-only treatment	3.9 (1.2)	3.8 (1.2)	3.9 (1.2)
Check with patient about naloxone	4.5 (0.8)	4.4 (0.8)	4.5 (0.7)
Willingness to prescribe preexposure prophylaxis	3.1 (1.2)	3.2 (1.3)	3.1 (1.1)
Willingness to refer to syringe service program	3.4 (1.2)	3.3 (1.3)	3.5 (1.1)

^a^ Scored on a 5-point Likert scale of 1 (extremely unlikely) to 5 (extremely likely).

### MANCOVA Results

The MANCOVAs examining clinical outcomes indicated a significant overall *F* test result for the chief concern condition (*F*_4,360_ = 41.80; *P* < .001) but a nonsignificant overall *F* test result for the patient race condition (*F*_4,360_ = 0.83; *P* = .51) and the interaction between the chief concern and patient race conditions (*F*_4,360_ = 1.17; *P* = .32) (Table 2). For each clinical outcome, a significant *F* test result for the chief concern condition was found for the items assessing willingness to treat the patient (*F*_4,363_ = 40.92; *P* < .001), desire to refer the patient to a specialist (*F*_1,363_ = 92.37; *P* < .001), and perceived likelihood of disease stabilization (*F*_1,363_ = 8.01; *P* = .005). The *F* test result for the item assessing the amount of time participants were willing to spend with the patient was not significant (*F*_1,373_ = 3.36; *P* = .07) (Table 3). Pairwise comparisons indicated that participants in the OUD condition were significantly less willing to treat the patient (mean [SE], 3.6 [0.1]) than those in the type 2 diabetes condition (mean [SE], 4.4 [0.1]) (*P* < .001), and those in the OUD condition were significantly more likely to refer the patient to a specialist (mean [SE], 4.4 [0.1]) compared with those in the type 2 diabetes condition (mean [SE], 3.3 [0.1]) (*P* < .001). Participants in the OUD condition rated the likelihood of disease stabilization significantly higher (mean [SE], 3.6 [0.1]) than those in the type 2 diabetes condition (mean [SE], 3.3 [0.1]) (*P* = .005) (Table 4).

**Table 2.** Multivariate Analysis of Covariance Results for Chief Concern and Race Associated With Outcomes

Covariate	Clinical outcomes, *F*_4,360_	Attitudinal outcomes, *F*_2,362_	Attributes for disease, *F*_6,358_
Physician	1.70	1.25	1.60
Nurse practitioner	1.21	1.55	0.78
Respondent sex	2.48^a^	0.97	1.80
Respondent race	0.47	1.24	4.05^b^
Federally Qualified Health Center	0.68	0.46	1.19
Positive contact	24.87^b^	55.01^b^	3.25^c^
Chief concern condition	41.80^b^	12.85^b^	16.32^b^
Race condition	0.83	0.29	1.79
Chief concern × race	1.17	0.67	0.64

^a^ *P* < .05.

^b^ *P* < .001.

^c^ *P* < .01.

**Table 3.** Analysis of Covariance Results for Chief Concern and Race Associated With Clinical Outcomes

Covariate	Willingness to treat, *F*_1,363_	Refer to specialist, *F*_1,363_	Time for visit, *F*_1,363_	Remission, *F*_1,363_
Physician	0.61	3.52	2.52	0.02
Nurse practitioner	1.24	0.06	2.59	0.36
Respondent sex	0.29	0.10	7.09^a^	1.83
Respondent race	0.34	1.37	0.06	0.14
Federally Qualified Health Center	0	1.44	0.66	0.68
Positive contact	25.86^b^	19.03^b^	5.54^c^	56.48^b^
Chief concern condition	40.92^b^	92.37^b^	3.36	8.01^a^
Race condition	0.30	3.15	0.02	0.13
Chief concern × race	0.77	0.33	0.09	3.12

^a^ *P* < .01.

^b^ *P* < .001.

^c^ *P* < .05.

**Table 4.** Pairwise Comparisons of Response Scores by Study Condition

Outcome	Response score, mean (SE)^a^			
	Chief concern		Patient race	
	Opioid use disorder	Type 2 diabetes	Black	White
Clinical				
Willingness to treat	3.6 (0.1)	4.4 (0.1)^b^	4.0 (0.1)	4.0 (0.1)
Refer to specialist	4.4 (0.1)	3.3 (0.1)^b^	3.8 (0.1)	4.0 (0.1)
Time for visit	3.9 (0.1)	3.7 (0.1)	3.8 (0.1)	3.8 (0.1)
Remission	3.6 (0.1)	3.3 (0.1)^c^	3.5 (0.1)	3.5 (0.1)
Attitudinal				
Controllability	2.0 (0.1)	2.4 (0.1)^b^	2.2 (0.1)	2.2 (0.1)
Empathy	3.3 (0.1)	3.0 (0.1)^c^	3.2 (0.1)	3.1 (0.1)
Covariate				
Positive contact	3.0 (0.1)	4.0 (0.1)^b^	3.4 (1.1)	3.4 (1.0)

^a^ Scored on a 5-point Likert scale of 1 (extremely unlikely) to 5 (extremely likely).

^b^ *P* < .001.

^c^ *P* < .01.

Results for the overall MANCOVA examining empathy and perceived controllability indicated a significant association with the chief concern condition (*F*_2,362_ = 12.85; *P* < .001) but no association with patient race (*F*_2,362_ = 0.29; *P* = .75) and no significant interaction between chief concern and patient race (*F*_2,363_ = 0.67; *P* = .51). An examination of the *F* test results for the 2 dependent variables revealed a significant association with the chief concern condition on both perceived controllability (*F*_1,363_ = 21.98; *P* < .001) and empathy (*F*_1,372_ = 8.55; *P* = .004) (Table 5). The pairwise comparisons indicated that participants scored higher on perceived controllability in the type 2 diabetes condition (mean [SD], 2.4 [0.1]) than the OUD condition (mean [SD], 2.0 [0.1]) (*P* < .001). Moreover, participants scored higher on empathy in the OUD condition (mean [SD], 3.3 [0.1]) than the type 2 diabetes condition (mean [SD], 3.0 [0.1]) (*P* = .004) (Table 4). Results for the final model, which examined the 6 attributions for the chief concern as the dependent variables are available in the eAppendix and eTables 1 and 2 in Supplement 1.

**Table 5.** Analysis of Covariance Results for Chief Concern and Race Associated With Attitudinal Outcomes

Covariate	Controllability, *F*_1,363_	Empathy, *F*_1,363_
Physician	2.10	0.09
Nurse practitioner	1.14	1.30
Respondent sex	1.83	0.40
Respondent race	2.45	0.02
Federally Qualified Health Center	0.04	0.93
Positive contact	23.79^a^	102.90^a^
Chief concern condition	21.98^a^	8.55^b^
Race condition	0.16	0.31
Chief concern × race	1.31	0.01

^a^ *P* < .001.

^b^ *P* < .01.

## Discussion

This cross-sectional study assessed support for treating OUD within primary care, including with MOUDs, and compared PCP willingness to treat patients with OUD vs type 2 diabetes. Using an experimental design, we found that participants were more willing to treat patients with type 2 diabetes and more likely to refer patients with OUD to a specialist, even though they rated the likelihood of disease stabilization higher for patients with OUD. Primary care professionals may perceive that OUD is more complicated to treat than other chronic conditions, despite there being a relatively straightforward treatment available.^28^

Furthermore, participants viewed type 2 diabetes as being more within the patient’s control compared with OUD and expressed more empathy for the patient with OUD, which is somewhat surprising given well-documented stigma associated with OUD.^8,29,30,31^ Although diabetes stigma is also well documented,^32^ it is possible that type 2 diabetes may be perceived as a lifestyle choice given our findings that this diagnosis was perceived as being more within the patient’s control. The American Society on Addiction Medicine and the National Institute on Drug Abuse define addiction, including OUD, as a chronic, relapsing brain disease.^33^ Health care professionals who have received education using this definition may be less likely to perceive OUD as controllable and may have more empathy for people with OUD. Although there is growing evidence of the complex interaction between genetics and environmental factors,^34^ including lifestyle factors^35^ and social determinants of type 2 diabetes,^36^ some health care professionals may still perceive type 2 diabetes as primarily within a patient’s control.

We also explored whether patient race was associated with treatment decisions. Interestingly, our results indicated that the clinical outcomes, attitudinal outcomes, and attributions were consistent regardless of the patient’s race, which suggests that our participants were similarly willing to treat patients, regardless of patient race. It is important to emphasize that there is a substantial evidence base documenting racial disparities in the treatment of both OUD and type 2 diabetes.^22,37,38^

Our findings could inform future interventions to improve the treatment of OUD in primary care settings. Previous interventions to increase MOUD prescribing have focused on improving attitudes toward MOUDs and the clinical skills to prescribe them.^39,40,41^ These interventions may not address perceived compatibility of OUD treatment with primary care. One promising strategy is to provide implementation support that aligns OUD treatment, including MOUDs, with the chronic disease model in primary care. For example, emphasizing the role of long-term medication management for conditions such as hypertension or type 2 diabetes could help align OUD treatment with the existing primary care infrastructure.

Given the substantial amount of chronic disease management in primary care, participants may have preferred referring patients with OUD to specialist care due to time constraints.^5,42,43^ It is also possible that PCPs do not feel confident or believe that they have the appropriate training or experience to treat OUD. Our findings suggest that attitudes such as perceived controllability and empathy might matter less than having previous experience treating a specific condition. We found vastly different baseline levels of positive contact with OUD vs type 2 diabetes. Future training interventions should emphasize facilitating positive contact with patients with OUD and peer PCPs who treat OUD within primary care as a potential implementation strategy.^44^

Although referral to specialist care is indicated in specific cases, addiction medicine specialists and psychiatrists are scarce in many regions of the US, especially rural areas.^45,46^ Encouraging PCPs to deliver OUD treatment, especially providing evidence-based MOUDs, is critical. Our findings suggest only a moderate willingness to use MOUDs. Participants were more likely to recommend abstinence-based residential treatment, which does not effectively prevent overdose or associated morbidities.^47,48,49^ Additional clinical training on the effectiveness of MOUDs is warranted.

### Limitations

This study has important limitations and suggests opportunities for future research. Social desirability bias may have influenced our results, given that PCPs were asked to self-report their treatment decisions. Future research is necessary to assess actual treatment behaviors among PCPs. Furthermore, we used an expanded definition of primary care to capture clinicians who are likely to treat OUD. Approximately 10% of the sample included clinicians who are unlikely to manage diabetes (eg, psychiatry, pain management, addiction medicine, infectious disease), which may have influenced the results. Additionally, participants all came from the state of Ohio, which may limit the generalizability of findings. Future studies should determine whether willingness to treat OUD within primary care varies across geographic regions of the US. We also note that although we focused on primarily investigating differences in beliefs and behavioral intentions toward patients with OUD and type 2 diabetes, we did not directly examine behaviors or clinical outcomes. We also could not identify whether the differences observed here translate into clinically significant differences in treatment decisions. Finally, given our sampling method, we were unable to estimate the response rate, which might also influence the generalizability of our findings.

## Conclusions

This cross-sectional study found that a substantial primary care implementation gap remains for evidence-based MOUD treatment. We found that PCPs viewed a different chronic condition, type 2 diabetes, as more complicated to treat, viewed patients with type 2 diabetes as more responsible for their diagnosis, and had less empathy toward these patients. Nonetheless, PCPs were more willing to treat type 2 diabetes in primary care and more likely to refer patients with OUD to a specialist. Future implementation work is needed to determine whether addressing the implementation climate for treating OUD within primary care, especially perceptions that this setting is compatible with OUD treatment, could improve treatment access and OUD outcomes.

## Notes

Supplement 1. **eAppendix.** Analyses of Illness Attributions **eTable 1.** ANCOVA Results of Chief Concern and Race Associated With Disease Attributes **eTable 2.** Means and SEs for Disease Attributes by Condition

Supplement 2.
Data Sharing Statement
