# Supplementary material for: Health Care Professional Willingness to Treat Opioid Use Disorder vs Type 2 Diabetes in Primary Care
Source: JAMA Netw Open. 2025 Sep 30;8(9):e2534680. doi: 10.1001/jamanetworkopen.2025.34680 (PMC12485638; doi:10.1001/jamanetworkopen.2025.34680)
Supplement: Supplement 1. — eAppendix. Analyses of Illness Attributions eTable 1. ANCOVA Results of Chief Concern and Race Associated With Disease Attributes eTable 2. Means and SEs for Disease Attributes by Condition [file jamanetwopen-e2534680-s001.pdf]

Supplemental Online Content

Dhanani LY, Brook D, Hall OT, Chang JE, Franz B. Primary care professional willingness to treat opioid use disorder vs type 2 diabetes. *JAMA Netw Open*. 2025;8(9):e2534680. doi:10.1001/jamanetworkopen.2025.34680

eAppendix. Analyses of Illness Attributions
eTable 1. ANCOVA Results for Chief Concern and Race Associated With Disease Attributes
eTable 2. Means and SEs for Disease Attributes by Condition

This supplemental material has been provided by the authors to give readers additional information about their work.

© 2025 Dhanani LY et al. *JAMA Network Open*.

eAppendix. Analyses of Illness Attributions

Illness Attributions:
We measured perceived attributions of Type 2 diabetes and OUD using 6 items. Participants were asked how likely 6 different factors contributed to each illness: personal choices, genetics or a disposition, prior medical history, a mental health disorder, socioeconomic/environment factors, and demographic/cultural factors.

Results:

Results indicated a significant overall F-test for chief complaint condition (F (6, 358) = 16.32, p < .001), but nonsignificant overall F-tests for patient race (F (6, 358) = 1.79, p = .100) and for the interaction between chief complaint and patient race (F (6, 358) = .64, p = .697) (Supplemental Table 1). Results for each of the specific attributes then revealed that there was a significant F-test for the chief complaint condition for the following attributes: personal choices (F (1, 363) = 7.92, p = .005), a mental health disorder (F (1, 363) = 51.98, p < .001), socioeconomic/environmental factors (F (1, 363) = 9.35, p = .002), and demographic/cultural factors (F (1, 363) = 5.43, p = .020). The pairwise comparisons indicated that people in the OUD condition were significantly less likely to perceive the condition to be a result of personal choices (M = 3.74, SE = .07) as compared to those in the Type 2 diabetes condition (M = 4.03, SE = .07, p = .005). Further, those in the OUD condition were significantly more likely to perceive the condition to be a result of a mental health disorder (M = 4.08, SE = .07) as compared to those in the Type 2 diabetes condition (M = 3.23, SE = .08, p < .001). Those in the OUD condition also scored significantly lower on socioeconomic/environmental factors (M = 4.25, SE = .05) as compared to those in the Type 2 diabetes condition (M = 4.51, SE = .06, p = .002). This was also the case for demographic/cultural factors, with those in the OUD condition scoring lower (M = 3.96, SE .06) than those in the Type 2 diabetes condition (M = 4.19, SE = .07, p = .020) (Supplemental Table 2).

© 2025 Dhanani LY et al. *JAMA Network Open*.

eTable 1. ANCOVA Results for Chief Concern and Race Associated With Disease Attributes

	Fs					
	Personal choice	Genetics	Medical history	Mental health	Socioeconomic /environmental	Demograph ic/cultural
Physician	.98	2.08	.44	4.73*	.02	.03
Nurse practitioner	.47	.13	.68	2.49	.03	1.32
Respondent gender	.16	7.59**	.01	.67	.94	.28
Respondent race	1.21	12.47***	.69	2.75	.24	2.28
FQHC	1.39	.53	1.57	.03	.98	2.83
Positive contact	6.19*	9.43**	.00	.28	1.56	.02
Chief complaint condition	7.92**	3.45	.86	51.98***	9.35**	5.43**
Race condition	.10	.20	5.30*	1.08	1.44	.26
Chief complaint x race	.49	1.69	.00	.57	1.88	.11

Note: * $p < .05$, ** $p < .01$, *** $p < .001$.

© 2025 Dhanani LY et al. *JAMA Network Open*.

eTable 2. Means and SEs for Disease Attributes by Condition

	Mean (SE)			
	Chief Complaint		Patient Race	
	OUD	Type 2 Diabetes	White	Black
Personal choices	3.74 (.07)	4.03 (.07)**	3.90 (.06)	3.87 (.07)
Genetics	3.98 (.06)	4.17 (.07)	4.06 (.06)	4.10 (.06)
Prior medical history	4.16 (.06)	4.07 (.07)	4.01 (.06)	4.21 (.06)*
Mental health disorder	4.08 (.07)	3.23 (.08)***	3.60 (.07)	3.71 (.07)
Socioeconomic/environmental	4.25 (.05)	4.51 (.06)**	4.43 (.05)	4.34 (.05)
Demographic/cultural	3.96 (.06)	4.19 (.07)*	4.10 (.06)	4.06 (.06)

Note: * $p < .05$, ** $p < .01$, ***$p < .001$.

© 2025 Dhanani LY et al. *JAMA Network Open*.
