# Supplementary material for: Health Care Professional Willingness to Treat Opioid Use Disorder vs Type 2 Diabetes in Primary Care
Source: JAMA Netw Open. 2025 Sep 30;8(9):e2534680. doi: 10.1001/jamanetworkopen.2025.34680 (PMC12485638; doi:10.1001/jamanetworkopen.2025.34680)
Supplement: Supplement 2. — Data Sharing Statement [file jamanetwopen-e2534680-s002.pdf]

Data Sharing Statement

Data
Data available: Yes
Data types: Deidentified participant data, Data dictionary
How to access data: Data will be shared upon reasonable request to franzb@ohio.edu
When available: With publication

Supporting Documents
Document types: Statistical/analytic code
How to access documents: Statistical code will be shared upon reasonable request to franzb@ohio.edu
When available: With publication

Additional Information
Who can access the data: Data will be made available to researchers whose proposed use of the data has been approved.
Types of analyses: Data will be made available for any purpose.
Mechanisms of data availability: Data will be available after receipt of a signed data access agreement.
Any additional restrictions: N/A
